# Supplementary material for: “An app is just available at all times”—the process and outcomes of converting the Georgia Tuberculosis Reference Guide into a mobile application
Source: PLoS One. 2024 May 16;19(5):e0298758. doi: 10.1371/journal.pone.0298758 (PMC11098468; doi:10.1371/journal.pone.0298758)
Supplement: Attachment — Submitted filename: Review-Reply_PloS.docx [file pone.0298758.s001.docx]

Response to Email Issues

Dear Editor,

Thank you for reviewing our submission to PloS One. Please find below the responses to the comments outlined in the email.

1. We have listed the affiliations of all the authors in the updated manuscript

2. We did not seek IRB approval for this study as this was considered a QA/QI project by our institution. The study is focused on improving local practice and the findings are not meant to be generalizable beyond disseminating best practices. Please see the information from the Emory [eIRB website](https://irb.emory.edu/guidance/getting-started/review.html) for your reference.

|  | [Does My Project Need IRB Review? \| Emory University \| Atlanta GA](https://irb.emory.edu/guidance/getting-started/review.html)  property=  irb.emory.edu |
| --- | --- |

"Typically, QA/QI projects are particularly focused on improving the performance of an institutional practice in comparison with an established standard or goal. They are focused on a local practice and consequently limit their scope to the specific institution. The results of the project are not *intended* to apply to anyone beyond the scope of the project, and conclusions are drawn only in relation to the particular practice at the institution. In other words, if the results of the project are shared outside the institution (i.e. published or presented), then it would only be for the purposes of sharing a successful improvement in practice; other institutions could then interpret the results and draw their own conclusions, but the key is that the institution conducting the QA/QI project is not drawing broad conclusions and is not using their participants as a representative sample."

We have attached the communication with the IRB certifying that we do not need an IRB. If an approval from the IRB is required even as a Quality Improvement project, please let us know and we'd be happy to obtain it.

3. Informed consent was not obtained as described the response to point 2

4. We have attached the completed Clinical Studies Checklist to this email. As per the definition of Human Subjects Research, this study is IRB-exempt and thus no Human Subjects Research was performed.
